# Antidotal Properties of Permanganate of Potassium

**Published:** 1899-12

**Authors:** 


### Antidotal Properties of Permanganate of Potassium.

The ease with which permanganate of potassium gives up its oxygen when brought into contact with oxidizable substances renders it of extreme value as an antidote in respect of many, if not indeed of most, of the alkaloids. It is admittedly the most efficacious agent we possess in cases of poisoning by opium and its alkaloids, and it has been estimated that a given weight of the salt will neutralize a similar quantity of morphine. This valuable property, however, is not limited to alkaloids of the morphia series. Stokvis recommends it as an antidote in poisoning by phosphorus, which it transforms into orthophosphoric acid. Autail has shown that it neutralizes the toxic properties of oxalic acid, the cyanides, strychnine, colchicine and muscarine. Lacerda long since recognized it in the treatment of serpent bites, while Durante and Giodano found it equally of service in attenuating the symptoms resulting from the bites of insects. Somewhat later Hugouneng demonstrated its antidotal action in respect of caffeine, cocaine and alkaloids of the atropine group, and still more recently Paratore has shown that curare, nicotine and aconitine are also destroyed thereby. Comparative experiments with the permanganate show it to be far more trustworthy than tannin or iodine in the treatment of strychnine poisoning, whether employed hypodermically or for washing out the stomach. It would seem, indeed, that the permanganate is the agent par excellence to which the physician ought to have recourse whenever he is confronted with a case of suspected alkaloidal poisoning. In view of these marked antidotal properties, it is possible that the administration of permanganate might prove of service in the frequent cases of ptomaine poisoning, the lethal agent in these cases being presumably alkaloids—alkaloids, moreover, which are peculiarly susceptible of oxidation.—*Med. Press and Circular.*

Reception to the Medical Students.—The faculty of the medical and dental departments of the University of Maryland, Baltimore, tendered a reception to nearly fivehundred students in the lecture room of the law department, February 22d.

